# BeautyNet: Joint Multiscale CNN and Transfer Learning Method for Unconstrained Facial Beauty Prediction

**DOI:** 10.1155/2019/1910624

**Published:** 2019-01-28

**Authors:** Yikui Zhai, He Cao, Wenbo Deng, Junying Gan, Vincenzo Piuri, Junying Zeng

**Affiliations:** ^1^School of Information Engineering, Wuyi University, Jiangmen, China; ^2^Department of Computer Science, Universita' Degli Studi di Milano, Crema, Italy

## Abstract

Because of the lack of discriminative face representations and scarcity of labeled training data, facial beauty prediction (FBP), which aims at assessing facial attractiveness automatically, has become a challenging pattern recognition problem. Inspired by recent promising work on fine-grained image classification using the multiscale architecture to extend the diversity of deep features, BeautyNet for unconstrained facial beauty prediction is proposed in this paper. Firstly, a multiscale network is adopted to improve the discriminative of face features. Secondly, to alleviate the computational burden of the multiscale architecture, MFM (max-feature-map) is utilized as an activation function which can not only lighten the network and speed network convergence but also benefit the performance. Finally, transfer learning strategy is introduced here to mitigate the overfitting phenomenon which is caused by the scarcity of labeled facial beauty samples and improves the proposed BeautyNet's performance. Extensive experiments performed on LSFBD demonstrate that the proposed scheme outperforms the state-of-the-art methods, which can achieve 67.48% classification accuracy.

## 1. Introduction

Assessing facial beauty is a natural action for people, as an attractive one has more advantages in social life. Psychology research revealed that apart from cultural and contemporary factors, what is more important is that one's evaluation is often influenced by various factors such as clothing, hairstyle, social status, personal feelings, and others' evaluation. As the saying goes, “Beauty lies in the eyes of the beholder,” facial beauty is an abstract concept, and each person's definition of beauty is different. With the development of artificial intelligence, studies [1–3] indicate that facial attractiveness can be learned by machine learning using data-driven methods. Recently, facial beauty prediction becomes an emerging research area due to many potential applications, such as aesthetic surgery planning [1], cosmetic recommendation [2], and face-based pose analysis [3].

Deep learning has provided state-of-the-art performance in many tasks in recent years, ranging from computer vision [4] to natural language processing [5]. Contrary to traditional machine learning methods, in which features are chosen manually and extracted through instructed algorithms, deep learning networks automatically discover increasingly higher level features from data. For CNNs, the outputs of the last convolutional layers encode the semantic information of specific task, which are robust to significant appearance variations [6]. However, their spatial resolution is too coarse to preserve the texture information of the image, which is extremely important for facial beauty prediction task. For the facial beauty prediction task, which has large interclass variance, the existing CNN methods exploiting the features extracted from the last output layer may be insufficient. Multiscale deep features [7] can extend the generalization of the features represented, which fuse different layers' feature maps together. The multiscale architecture has a large number of tunable parameters as compared to others, and it covers features at different resolutions and scales, which could achieve higher performance [8].

As we all know, effective training of neural networks requires abundant data. However, in the real world, facial beauty data with labels are often scarce, and it is expensive to obtain sufficient labeled facial beauty samples directly. The deficiency of labeled facial beauty prediction data may lead to overfitting on the training stage and further may result in poor generalization on the test stage. To tackle the data deficiency problem for such a small database task, the utilization of transfer learning strategy before the training of the target domain may be a good solution.

Transfer learning is utilized to improve a network learning ability from target domain by transferring information from source domain [9]. When the sample size of the target domain is too small to support the training of CNN, the use of transfer learning can provide a fine initialization state, which benefits further training and is much better than initializing the whole network randomly. Transfer learning parameters can give the network more relevant information and alleviate the phenomenon of model overfitting caused by the insufficiency of the training database, which will extremely improve the performance of the network [10].

**Figure 1:**
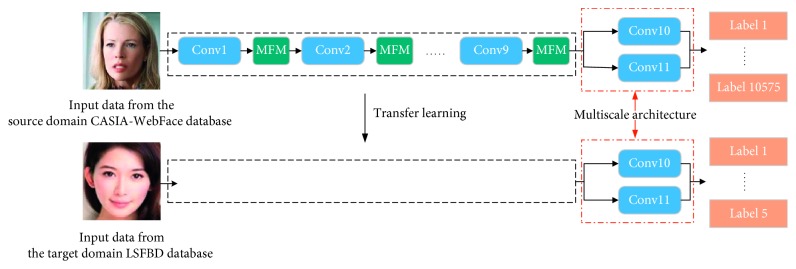
The whole framework of this paper.

In this paper, BeautyNet for unconstrained facial beauty prediction is proposed. BeautyNet adopted a multiscale architecture that can produce more discriminative and robust deep features. Then, in order to mitigate the computational burden of the multiscale structure, we adopted the MFM (max-feature-map) activation function to replace the common activation function. Compared to other traditional activation functions, such as ReLU, MFM activation function has a sparse gradient and compact representation simultaneously, which can extremely lighten the model and speed the network convergence. Finally, we adopted transfer learning to get a better initialization state for facial beauty prediction task, which utilized face recognition database as the source domain and facial beauty database as the target domain. Experimental results shows that transfer learning could perform much better. The whole framework of this paper is shown in Figure 1.

Our major contributions can be summarized as follows:

- A multiscale CNN architecture named BeautyNet is designed specifically for FBP task. The BeautyNet consists of basic convolution layers and a multiscale architecture. The deep features which contain semantic and texture information simultaneously, extracted deep features from the proposed BeautyNet, are suitable for our task.
- MFM (max-feature-map) activation function is presented here to achieve discriminative beauty feature. Compared to other traditional activation functions, MFM has a sparse gradient and compact representation simultaneously, which could not only lighten the model and fasten the convergence but also benefit the performance.
- Transfer learning strategy is also incorporated to alleviate the overfitting problem for unconstrained facial beauty prediction task with limited labeled database. The parameters of the pretrained model on large-scale face recognition database are exploited. For FBP task, some part parameters of the pretrained model were transferred and further trained on the BeautyNet. Experimental results show that transfer learning strategy can significantly improve its performance.

The remainder of this paper is organized as follows. In Section 2, we review the related work of facial beauty prediction, multiscale CNNs, and transfer learning.  Section 3 presents the details of the proposed BeautyNet and MFM activation function.  Section 4 proposes transfer learning method and gives its details. Experimental results and analysis are presented in Section 5. Finally, Section 6 concludes this paper.

## 2. Related Work

In this section, we will discuss related work in facial beauty prediction, multiscale CNN, and transfer learning.

### 2.1. Facial Beauty Prediction

Traditional facial beauty prediction focuses on a geometry-based method. For geometry-based methods, firstly, meaningful feature points from face images are extracted manually; then, geometric distances and ratio vectors between feature points are computed; finally, the feature vectors will be used for machine learning. Mao et al. [11] first proposed a method of automated facial beauty prediction, which presented a simple but effective feature extractor; then, the extracted geometric feature was used to train the SVM (support vector machine). Zhang et al. [12] adopted a normalized face and mapped it onto a facial shape space, then quantitatively analyzed the effect of facial geometric to overcome the transformation influence. Gunes et al. [13] computed the ratios of different facial components as features for facial beauty assessment. Schölkopf et al. [14] computed the distances and slopes of these landmarks. The extraction of geometry features depended on the detection of face landmark in the preprocessing stage, and the accuracy of landmark detection could directly affect the performance of facial beauty prediction. Therefore, geometry-based methods could achieve good prediction results for frontal face with constraint experiment environment, which could locate face landmark accurately. However, it cannot achieve satisfactory results in unconstrained facial beauty prediction, while the landmark detection may be seriously affected by many factors, such as illumination, occlusion, and blurring. To avoid heavily manual intervention and burden landmark in geometry-based methods, and take advantage of large data, we established a large database named LSFBD in [15], and multiscale apparent features are utilized for facial beauty prediction. In this paper, we continue to explore the potential of CNN on the facial beauty prediction task based on the LSFBD.

Recently, deep learning has been demonstrated to be a promising area of research in machine learning. Some researchers have used deep learning to predict facial beauty and achieved satisfactory results. Gray et al. [16] directly employed images to CNN network for learning, without marking the key points of the images, and realized automatic facial beauty prediction. Gan et al. [17] adopted deep self-taught learning method to extract facial beauty features without depending completely on artificial feature selection and obtained human-like performance. Xu et al. [18] carefully constructed a convolution neural network (CNN) for facial beauty prediction, which cascaded various inputting channels, such as the original RGB face image, the detail layer image, and the lighting layer image. Chen et al. [19] fused rule-based features, global features, and local descriptors and then reduced the dimension of feature and selected it, which could serve as a competitive prediction method. Xu et al. [20] proposed a psychologically inspired convolutional neural network (PI-CNN) to achieve facial beauty prediction, which facilitated both the facial beauty representation learning and predictor training. Zhang et al. [21] combined several low-level face representations and high-level features to predict facial beauty. Although contemporary CNN models obtained significant performance improvement, they only exploited the features extracted from the last output layer for classification, which may be insufficient for facial beauty prediction task.

### 2.2. Multiscale CNNs

Multiscale representation is a classic concept in computer vision and has been widely used in visual recognition [22], edge detection [23], and person reidentification [24]. Typical approaches train a CNN using features extracted from a single output layer. Instead, multiscale CNN can train an output predictor using features extracted from multiple layers, and this special structure has more advantages.

Yang and Ramanan [25] used DAG-CNNs (directed acyclic graph) to learn multiscale deep features and showed the effective of both coarse and fine-grained classification tasks. Li and Yu [26] utilized multiscale segmentation instead of single segmentation and then computed the visual saliency to boost the visual recognition performance. Ma et al. [27] combined the feature representations of last convolutional layers and early convolutional layers to complement each other and improved the accuracy and robustness of visual target tracking. Zhao et al. [28] adopted multiscale feature maps to obtain richer information, and the proposed SMSC (selected multiscale convolution feature) obtained more compact deep representations. Faraji et al. [29] presented a multiscale method on the maximum response filter bank and the gradients of faces, mitigating the effect of illumination variations in face recognition systems.

For a CNN, the top layer encodes the high-level features, such as semantic similarity; and the bottom layer encodes the low-level feature, such as texture similarity. For facial beauty prediction task, semantic information and texture information are both critical. Hence, in this paper, we proposed a multiscale architecture for facial beauty prediction, which could combine both high-level and low-level features. Experimental results show that the multiscale structure could obtain more satisfying classification results.

### 2.3. Transfer Learning

**Figure 2:**
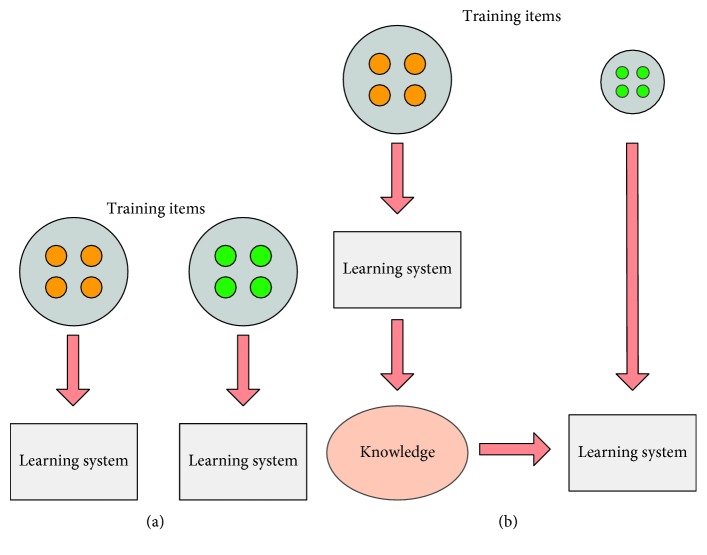
(a) Traditional machine learning vs. (b) transfer learning.

Transfer learning [9] is a simple but effective technique that can improve a network from one domain (target domain) by transferring parameters from an already trained related domain (source domain). It is essential to adjust the weight of data in the source domain for use in the target domain discriminately. Since the pretrained model already contains a lot of basic information, transfer learning can achieve better performance than the scratch network. The difference between traditional machine learning and transfer learning is illustrated in Figure 2. Transfer learning can address the sample deficient problem of a small database, improving the model learning performance, which is desirable for our task.

Many research found transfer learning is truly beneficial. Lu et al. [30] designed a framework, SFTL (source free transfer learning), to improve the text classification performance. Zhao et al. [31] used active transfer learning to realize the cross-system recommendation. Zhu et al. [32] adopted transfer learning to improve the image classification. Yosinski et al. [33] verified that the transferred parameter from almost any number of layers can produce an improvement to target domain task even after fine tuning to the target database. Shelhamer et al. [34] transferred some contemporary classification network's parameters to a fully convolution network by fine tuning in the segmentation task. Shin et al. [35] examined when and why transfer learning from pretrained ImageNet could be useful for image recognition. Girshick et al. [36] adopted a transfer learning method to address the problem of inadequate model performance caused by the deficiency of training data for object detection performance.

In this paper, transfer learning method is utilized to obtain low-level features from the source domain and speed BeautyNet converge to an optimal solution. Extensive experiments proved that transfer leaning can alleviate the overfitting phenomenon to some extent, improving the final classification accuracy and the Pearson correlation coefficient of facial beauty prediction.

## 3. Network Architecture

In this section, we first introduce the multiscale architecture based BeautyNet, which could have more expressive features and less computation expenditure. Then, the compact MFM (max-feature-map) activation function was introduced, which can not only lighten the network but also accelerate the network convergence. Thus, BeautyNet could improve the model performance finally.

### 3.1. BeautyNet

For the facial beauty prediction task, with a large interclass variance, contemporary CNN models only exploit the features extracted from the last output layer, as classification may be insufficient. Multiscale CNN features have been widely used in visual recognition [37], object detection [38], and visual tracking [39] due to their diversity. For multiscale connection, the top convolution layers encode high-level beauty information of facial parts, and such representations are robust to significant appearance variations, while bottom layers can capture the low-level beauty detail information of facial images, such as the object shapes or parts which may be important for facial beauty prediction task. Fusing multiple layers output feature, the diversity of the deep features will lead the model to achieve a better classification performance.

In this paper, we proposed BeautyNet to further improve the facial beauty prediction performance. Based on BeautyNet, we extract deep features from multiple CNN layers; these high-level and mid-level features were fused together for the final classification.

**Figure 3:**
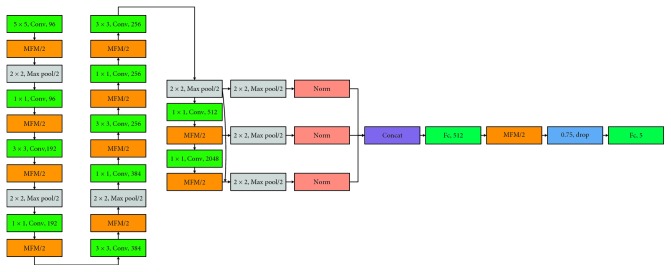
The architecture of BeautyNet.

The architecture of the proposed BeautyNet is illustrated in Figure 3, and the parameter setting is shown in Table 1. This CNN model is constructed by 11 convolution layers, MFM activation functions, 7 max-pooling layers, 3 normalization layers, 1 dropout layer, and 2 fully connected layers. The input image of the network is 120 × 120 RGB images from LSFBD. In the training stage, we also mirror and shuffle images. The MFM activation function and max pooling layer are utilized before convolutional layers. The Fc1 layer is a 512-dimensional facial beauty representation. And, the Fc2 layer serves as the input of Softmax cost function, and the number of output feature map set as the number of facial beauty categories.

The multiscale connection could achieve deep features with different resolutions and scales, render the network has more adjustable parameters, and extend the learning ability, leading BeautyNet to achieve the highest facial beauty prediction performance. Although the multiscale structure is valid, the multiscale connection will also bring much parameter calculations. Hence, in order to lighten the multiscale network, we also adopted MFM (max-feature-map) activation function instead of the common one, such as ReLU, which can obtain more sparse gradients and compact representation simultaneously. Owing to MFM's better characters, the model is lightened and the training convergence speed is faster. We compared the proposed model with the state-of-the-art methods, and experimental results validate its effectiveness.

Our contribution to the network structure is mainly embodied in the following three aspects:

1. The convolution kernels of the network in this paper only use three small values, 1 × 1, 3 × 3, and 5 × 5. The smaller convolution kernel size can effectively reduce the computation cost and obtain a lighten network.
2. The MFM activation function after each convolution layer can obtain not only sparse gradient but also compact feature representation. The number of input feature maps will reduce by half after the MFM activation function, greatly reducing the computation of network parameters. A suitable activation function will make the model converge faster.
3. Before the fully connection layer, we design a multiscale structure. The BeautyNet gives the highest performance because it has a large number of tunable parameters as compared to others and it covers features at different resolutions and scales.

### 3.2. Max-Feature-Map Activation Function

The activation function introduces nonlinear elements to CNN, making it a powerful nonlinear fitting capability. Nowadays, there are various activation functions available, such as Sigmoid, Tanh, and ReLU. These activation functions are well known because of robust optimization in network training, but they are also resented by researchers for their vulnerability to vanishing gradient. When the vanishing gradient phenomenon appears, the CNN training will be destroyed because of which the convergence of CNN will be slowed down or even fail.

To alleviate this problem, we adopted MFM (max-feature-map) activation function, which has a sparse gradient and compact representation simultaneously. It is worth noting that the MFM function is the combination of activation function and dimension reduction operation. MFM activation function divides the input feature map into two parts randomly, then compares the neuron of two parts, and preserves the maximum parts. Specially, given an input convolution layer *C* ∈ *R*^*h*×*w*×2*n*^, as is shown in Eq. (1), the MFM activation function can be written as follows:

(1) $$\begin{array}{c} f_{i,j}^{k}=\underset{1\leq k\leq n}{\text{max}}\left(C_{ij}^{k},C_{ij}^{k+n}\right), \end{array}$$

where the number of feature map in the input convolution layer is 2*n*, 1 ≤ *i* ≤ *h*, 1 ≤ *j* ≤ *w*. As is shown in Equation (1), the output *f* via MFM activation function belongs to *R*^*h*×*w*×*n*^.

According to Equation (2), the gradient of MFM activation function can be shown as

(2) $$\begin{array}{c} \frac{\partial f}{\partial \;C^{k^{\prime }}}=\left\{\begin{array}{cc} 1, & \text{if}\;\;C_{ij}^{k}\geq C_{ij}^{k+n},\\ 0, & \text{otherwise}, \end{array}\right. \end{array}$$

where 1 ≤ *k*′ ≤ 2*n* and

(3) $$\begin{array}{c} k=\left\{\begin{array}{cc} k^{\prime }, & 1\leq k^{\prime }\leq n,\\ k^{\prime }, & n+1\leq k^{\prime }\leq 2n. \end{array}\right. \end{array}$$

**Figure 4:**
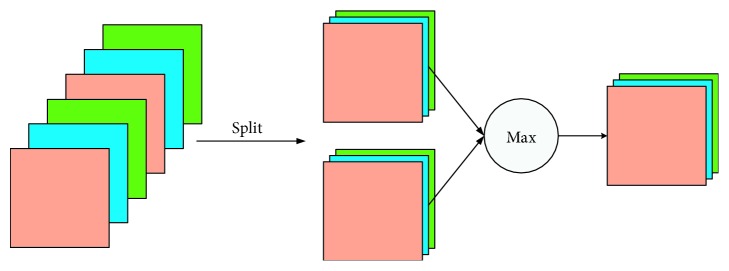
Operation performed by the max-feature-map activation function.

The MFM activation function can get sparse gradients, where 50% gradients values are 0. The input layer's feature maps were divided into two candidate neuron units A and B. The MFM activation layer is the maximum between A and B, forming new feature maps and output. MFM activation function which utilizes a statistics method can not only obtain a sparse gradient but also a compact representation and is important for classification tasks. Among them, sparse gradients can fasten the convergence of the model and compact representation can help reducing data dimensions while maintaining model performance. The structure of the MFM activation function is illustrated in Figure 4.

## 4. Transfer Learning

**Figure 5:**
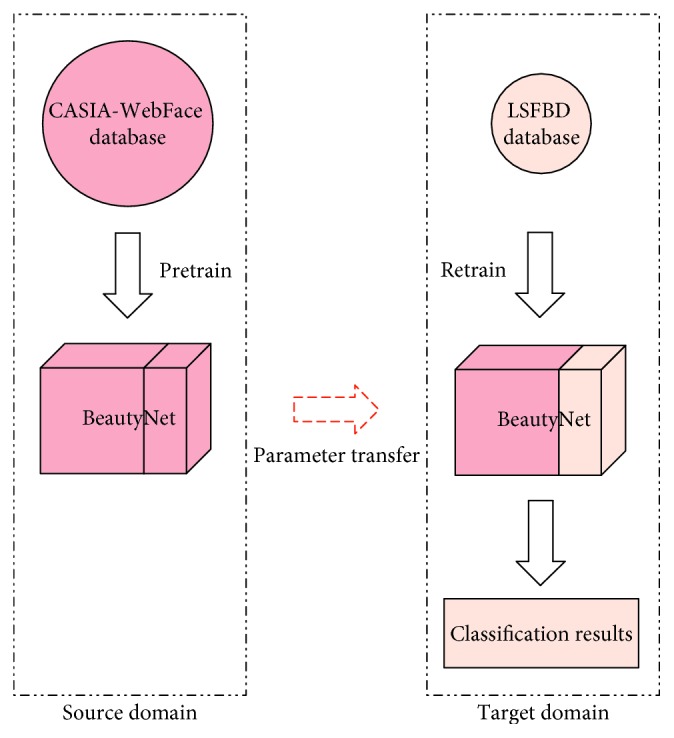
Transfer learning schematic diagram.

Deep convolution neural networks are successfully used in wide applications due to their ability to learn rich image representations. However, large amounts of data are required to learn these features. For facial beauty prediction database, the data amount is deficient, leading to the overfitting phenomenon. Since facial beauty prediction and face recognition tasks have different marginal probability distribution and the same feature space, the network's performance could be improved by transfer learning. Transfer learning is a method of transferring knowledge from a related domain to a new problem. Transfer learning strategy learns both low- and mid-level features from the transferred domain, and thus requires a little amount of data from the new domain to achieve higher performance. In this paper, we adopted the strategy of transfer learning to compensate the impact of small facial beauty database on the performance of BeautyNet. The experimental results indicate the effectiveness of transfer learning. The schematic diagram of transfer learning is illustrated in Figure 5.

### 4.1. Definition of Transfer Learning

Given a source domain *D*_S_ and a target domain *D*_T_, which correspond to learning task *T*_S_ and *T*_T_, the purpose of transfer learning is to improve the learning ability of target prediction function *f*(*T*(·)) in *D*_T_ using the knowledge of *D*_S_ and *T*_S_, where *D*_S_ ≠ *D*_T_ or *T*_S_ ≠ *T*_T_.

More specifically, for facial beauty prediction task, the source domain is defined as *D*={*F*, *P*(*X*)}, where *F*={*f*_1_, *f*_2_,…, *f*_*n*_} is a feature space with *n* dimensions, *f*_*i*_ is a feature, *X*={*x*_1_, *x*_2_,…, *x*_*n*_} is a facial beauty database, and *P*(*X*) is the marginal probability distribution of *X*. For a domain that is thought to be different, the feature spaces or marginal probability distribution is different. The task domain is defined as *T*={*y*, *P*(*y* | *X*)}, where *y* is the label space and *P*(*y* | *X*) is the classification model.

In this paper, we implement transfer learning as follows:

1. Firstly, we adopted the proposed network (BeautyNet, which only changes the output of the last fully connected layer for specific classification task) to train the face recognition tasks on CASIA-WebFace database, and through continuous parameter optimization, the net has obtained the state-of-the-art face recognition performance on the LFW database. This step is to help the model learn the facial features from a large-scale face database, which could contain the generalization ability of CNN and help the model to learn more discrimination deep features.
2. Secondly, for network parameter initialization of facial beauty prediction, we transfer the shallow layer's parameters, whereas other layers were randomly initialized; then, the parameters of the learned layer is frozen, and the hyperparameters of BeautyNet are retrained on LSFBD until the model converges to the optimal solution. Retraining the high-level features of the model is aimed at getting related features to our task. Specifically, when we transferred the parameters of conv6, we will shut the parameters update of the layer before it and only open the parameters update of the layer after it.
3. Finally, we refrozen the shallow layer and adopted the small learning rate to further fine tune the model, until model convergence stability. This step fine tunes the entire network parameters slightly, to make the model more suitable for the facial beauty prediction task, and obtained the best performance. Experimental results show performance improvement of the BeautyNet when transfer learning strategy is incorporated.

## 5. Experiments and Analysis

The experiments were configured with a desktop computer with an Inter3-6100, 3.70 GHz CPU, 16 GB RAM, and a single Nvidia GeForce GTX 1080 on a Windows 10 operating system. The training and testing of the proposed BeautyNet are based on the publicly available Caffe library [40].

### 5.1. LSFBD

LSFBD is a large-scale facial beauty database constructed by Zhai et al. [15], which is used in facial beauty prediction as a benchmark. LSFBD contains 20,000 labeled images, including 10,000 unconstrained male images and 10,000 unconstrained female images. In this database, each facial beauty image has a label, that is to say, “1” is extremely unattractive, “2” means unattractive, “3” means averages, “4” means attractive, and “5” is most attractive. The LSFBD images are selected from the website and contained a variety of variations, such as age, expression, angle, light, and occlusion. Moreover, the image quality is also diverse and uneven, which makes it difficult to predict the facial beauty.

**Figure 6:**
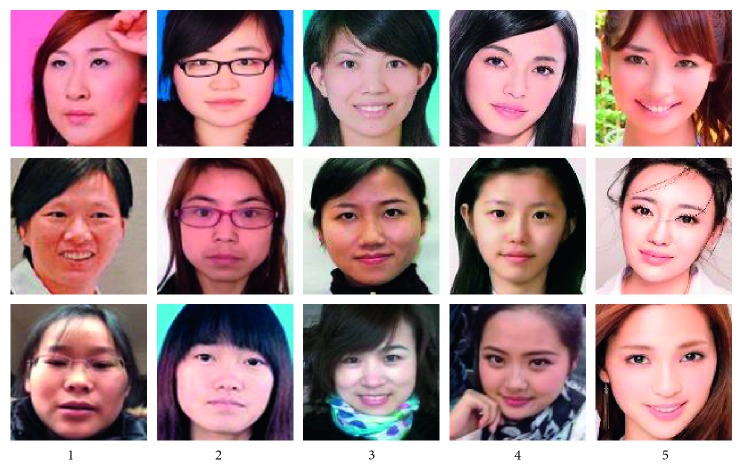
Some female examples of LSFBD.

**Figure 7:**
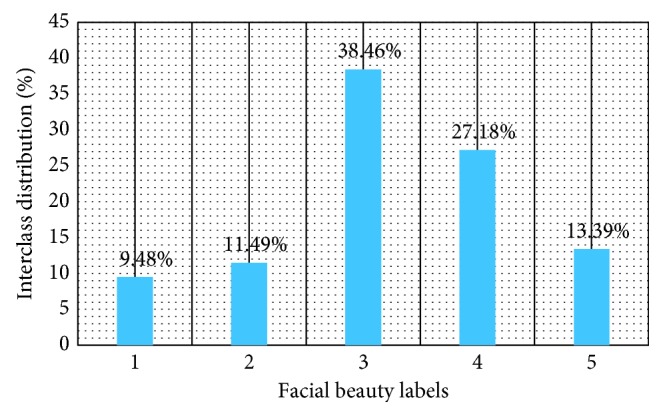
The distribution of LSFBD.

In this paper, we focused on predicting female beauty and only adopted 10,000 female images of LSFBD to verify the effectiveness of our overall framework for our facial beauty prediction task. For the convenience of subsequent description, in the following content, we still called the female part of LSFBD as LSFBD. Among LSFBD, category “1” contains 948 female images, category “2” contains 1,149 female images, category “3” contains 3,846 female images, category “4” contains 2,718 female images, and category “5” contains 1,339 female images, which contain 10,000 images totally.  Figure 6 shows some examples of LSFBD; each column of images belongs to the same category, and the degree of beauty increases in turn. The LSFBD distribution histogram is illustrated in Figure 7.

### 5.2. Configuration of Training Parameters

Training parameters are set as follows:

1. Prepare database, and divide the LSFBD into 9 : 1 as training and testing database, respectively. More specifically, the training set randomly selected almost 90% images from each class. The remaining images compose the testing database.
2. We select the initial learning rate of 0.001, adopt a batch size of 32, then initialize the tunable network parameters, and start the training of the network.
3. During the training stage, the learning rate is set as 10 times smaller when the test accuracy is no longer trending upwards, and training is continued until the test accuracy is no longer increasing.
4. The test results were obtained by balancing the model of test accuracy and stable loss.

### 5.3. The Impact of Network Depth

For deep neural networks, depth is an essential element of learning more abstract and robust representations. Numerous studies have demonstrated that deeper representations have more effective performance than insufficient ones. To evaluate the impact of network depth and find the most suitable layer sets, we compared the single-scale deep neural network with five, seven, nine, and eleven convolutional layers, denoted as NET-5, NET-7, NET-9, and NET-11, respectively. The experimental result is shown in Table 2.

It can be seen from NET-5 to NET-9 that with the increase of the depth, the classification performance of the proposed network is improved gradually, where the performance of classification from 64.36, 64.65, to 64.85, respectively, while Pearson's correlation coefficient is also increased from 79.61, 79.20 to 80.20. With the increase of the network depth, the performance showed a downward trend. The experimental results show that the network performance can be improved by increasing the network depth appropriately. However, for specific tasks and database size, when the network depth exceeds a certain range, overfitting phenomenon occurs and performance degrades. Hence, we choose nine convolution layers to construct the proposed network in this paper. Specifically, with two convolution layers contained in the multiscale structure, the proposed BeautyNet has a total of 11 convolution layers.

### 5.4. The Impact of Activation Function

The multiscale network structure adopted in this paper has improved the performance to some extent, but it brings the computational burden and makes the network difficult to converge. Therefore, this paper used MFM activation function to replace the traditional activation function, such as ReLU, Sigmoid, and Tanh, which could reduce the computational complexity of the model and speed up the convergence of the model. To verify the effectiveness of multiscale network structure on network performance via experiment, we removed the multiscale structure of BeautyNet and named this network as LightenNet.

MFM activation function has the effect of halving the number of feature maps; the amount of network parameter is reduced by half. For the fairness of experimental comparison, this section reduces the number of feature maps before every activation function of BeautyNet and LightenNet. In this section, we performed different activation functions to compare the parameters of the model, the size of the deep model, the speed of testing an image, and the classification accuracy. In addition, in order to further analyze the effect of different activation function, we visualized all the convolution layers of LightenNet and BeautyNet, and showed the visualization effectiveness of the first 25 feature maps of each convolutional layer.

From Table 3, it can be seen that for the same network, when different activation functions are used, the parameter calculation amount and the model size are consistent, which ensures the fairness of the comparison. When using LightenNet (the first four experiments), with ReLU activation function, the classification accuracy is 62.79; however, LightenNet cannot converge with Sigmoid or Tanh activation function; by using MFM activation function, LightenNet could converge and reach a 64.36 classification accuracy rate, which could perform better and faster than the other three activation functions. When adding multiscale structure (the last four experiments adopted BeautyNet), with ReLU or Tanh activation function, their classification accuracy all are 63.48; however, BeautyNet still cannot converge with Sigmoid activation function; by adopting MFM activation function, BeautyNet could reach a 64.84 classification accuracy rate, which could perform better and faster than the other three activation functions. It can be seen that for the same network, the MFM activation function has gained a greater advantage, showing its effectiveness; by adopting a multiscale structure, BeautyNet could achieve higher performance than LightenNet. Since the multiscale structure provides more facial beauty information, the BeautyNet with Tanh activation function could converge. MFM activation function divided the input feature map into two parts and output the maximum parts of it, which could reduce the nonsalient part of the feature map and remove the redundancy of feature representation.

**Figure 8:**
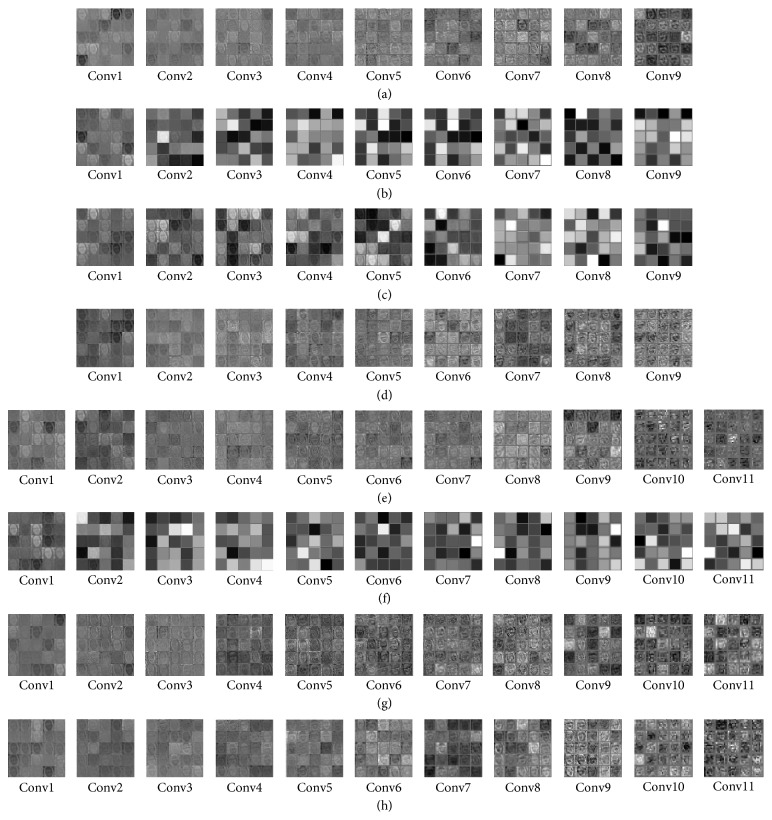
The visualization of the convolutional layers between different activation functions. LightenNet with (a) ReLU activation function, (b) Sigmoid activation function, (c) Tanh activation function, and (d) MFM activation function. BeautyNet with (e) ReLU activation function, (f) Sigmoid activation function, (g) Tanh activation function, and (h) MFM activation function.

To explore the specific effect of each convolution layers and analyze the situation of training, we visualized the feature map of LightenNet and BeautyNet with different activation function, as shown in Figure 8. For LightenNet, an intuitive phenomenon is that the feature maps of each convolutional layers using 8(a) ReLU and 8(d) MFM as activation functions are clear and have strong interpretability. However, it can be found that using 8(b) Sigmoid and 8(c) Tanh as activation functions, the feature maps after conv2 and conv6 are not interpretable, respectively. Since the network cannot learn useful information in the subsequent convolutional layer, the network cannot converge. By utilizing multiscale architecture with 8(e) Relu, 8(g) Tanh, and 8(h) MFM as activation function, the feature maps contain more information, leading the model to converge better. Specifically, by adopting multiscale architecture (adds more beauty information), the network with Tanh activation function could converge, which shows the effectiveness of multiscale structure. The performance of the network learning facial beauty prediction task under different activation functions is clearly in feature map shown as Figure 8. It can be seen that due to the sparse gradient of the MFM activation function, the compact representation of the network could avoid gradient disappearance phenomenon effectively, obtaining stronger learning performance.

### 5.5. The Impact of Multiscale Architecture

The existing facial beauty prediction method only extracts features from the final output layer for classification; however, semantic information and textural information are both important for our task. Thus, we adopted a multiscale architecture which could fuse low-level and high-level features to obtain deep features with different resolutions and scales. The diversity of the fused features could render with more robustness and stronger classification ability. The performance comparison results are shown in Table 4. Among them, LightenNet is the model of removing multiscale structure on BeautyNet.

From Table 4, compared with LightenNet, BeautyNet increased 0.48% of classification accuracy, and 0.79 of the Pearson correlation Ccoefficient, respectively. Although the performance of the network is improved after the incorporation of the multiscale structure, the computation of network parameters is also increased. Therefore, before the multiscale structure, MFM is adopted as the activation function behind each convolutional layer to compress the model parameters by at least half, thus greatly reducing the possibility of a long time to training and slow convergence caused by excessive calculation of model parameters.

### 5.6. Transfer Learning vs. Scratch

Researches show that for many deep neural networks trained on natural images all have one thing in common: the bottom layers learned the basic texture and color information, which appeared not to be specific to a particular database and tasks, so they could also be transferred to similar or different tasks to improve their performance. The top layer is adaptive to the specific task, and different tasks have different specific information. For small database task, to alleviate the overfitting phenomenon and further improve the model's prediction performance, we adopted transfer learning strategy for utilizing information in another source task.

Specifically, in this paper, the BeautyNet will be pretrained on the CASIA-WebFace database for face recognition task first, then parameters of this model were transferred for facial beauty prediction task, which could help BeautyNet obtain more related information, and finally the model will be retrained on LSFBD adaptive for facial beauty prediction task. To specifically observe the improvement of performance of facial beauty prediction task by transfer learning, we performed transferring parameters on all convolution layers of the network separately, among which multiscale structure was transferred as a whole to observe the effect of multiscale structure on network performance. For transferring parameters of each convolution layers, we retrained the model sufficiently to achieve the optimal performance.  Table 5 shows the specific results of the proposed network architecture validation on the LFW database which is trained in CASIA-WebFace database.  Table 6 shows the comparison between the scratch training and transfer learning training, and classification accuracy and Pearson's correlation coefficient are adopted for performance measurement. Among them, the larger the Pearson correlation coefficient, the greater the correlation between prediction labels and ground truth labels.

From Table 5, the proposed network could reach a 99.23% face recognition rate on the LFW database, which has outperformed the mainstream methods of DeepFace, DeepID2+, FaceNet, and VGG networks 1.46%, 1.96%, 52.13%, and 0.53%, respectively. Hence, the proposed network has learned enough facial detail information on the large-scale face database. For the facial beauty prediction task, the facial information learned from the face recognition task may be used to alleviate the impact of the lack of facial beauty data on the model performance.

From Table 6, we found that compared to the 64.84% accuracy rate of scratch, results via the proposed transfer learning are better, which is consistent with the previous results. The reason for this phenomenon is that no matter which layer is transferred, it will have more information before training the LSFBD than scratch, which is just trained by using the LSFBD. Among them, transferring the parameters of multiscale structure obtained the highest classification accuracy of 67.48%, which is 2.64% higher than the scratch one. At the same time, we also find that the accuracy of transferring Fc1 layer is only 65.53%, which is lower than transferring multiscale structure. This is because the convolution layers near the bottom of CNN network learn some texture and color information, and is not specific to a certain task. Therefore, transferring the weighting parameters of these layers maximum is useful to help the small database task to use the related information. However, the convolution layers near the top of CNN network learn the classification information, which should take appropriate adjustment for different tasks. Specifically, transferring the parameters of these layers may not be as straightforward as using random methods to initialize these layers. Hence, when we adopt transfer learning to improve the performance of the network, we should better choose the bottom layer parameters to transfer, rather than top layers.

For the Pearson correlation coefficient, transfer learning method could increase 80.20 of the scratch one to 83.54. Although transferring the parameters of the Fc1 layer obtained the highest Pearson correlation coefficient and showed a strong correlation between predict label and ground truth label, transferring the parameters of conv1, conv2, and conv3 layers obtained lower correlation. In general, the method of transfer learning was adopted to achieve a higher Pearson correlation coefficient than the scratch one.

### 5.7. Performance Comparison

In order to verify the effectiveness of the proposed BeautyNet, in this section, we compared its performance with that of other existing algorithms, and the comparison results are shown in Table 7. In Table 7, besides the proposed LightenNet, BeautyNet, and our previous method [15], there are four kinds of CNN models which reached outstanding performance in other research fields.

In Table 7, the first five experimental results are from [15], and the K-means method using multiscale images achieves the highest performance. This shows that the multiscale idea is beneficial to network performance. The next eight experimental data were adapted from NIN_Imagenet [45], DeepID2 [46], GoogLeNet [47], and VGG_CNN_S [48] network for facial beauty prediction, and shows the classification accuracy and Pearson correlation coefficient on the LSFBD. For the completeness of the experiment, we also added the performance of these four networks with transfer learning. The transfer learning method used here is consistent with that adopted by LighenNet and BeautyNet. For these four networks, the transfer learning method increases the classification accuracy by 4% to 5%, and the Pearson correlation coefficient by 2% to 3%, which shows that transfer learning could improve network performance. Due to the deep CNN architecture, however, deep network NIN_Imagenet, DeepID2, GoogLeNet, and VGG_CNN_S all achieve better performance than the method we proposed in [15]. Although these networks all achieved excellent performance in the mainstream recognition tasks, these network structures were too complex and deep for the facial beauty prediction tasks and are not specifically designed for our task, so the satisfying classification result was not achieved. Experimental results show that both LightenNet and BeautyNet with transfer learning are obviously superior to the state-of-the-art networks. BeautyNet designed in this paper has a simple structure and a moderate depth of convolution layers. The deep features used for the final classification combine semantic information and texture information simultaneously, with diversity and compactness, achieving better performance. It also inspired us; when using CNN to extract features, the depth of the network and related parameters should be adjusted according to the sample size of the training database in order to achieve better performance.

## 6. Conclusion

In this paper, we proposed a BeautyNet for unconstrained facial beauty prediction task. Different from the previous CNN model for facial beauty prediction, the multiscale which integrates the different scales features is presented here to obtain deep features, which is more effective for our task. In order to alleviate the computational burden of multiscale architecture, MFM activation function is adopted as a nonlinear unit for lightening the network and acceleration network convergence. Furthermore, transfer learning strategy is adopted to alleviate the overfitting phenomenon and achieved robust performance for unconstrained facial beauty prediction with limited labeled data. Extensive experiments performed on LSFBD show that the proposed scheme outperforms other state-of-the-art methods, which can obtain a 67.48% classification accuracy rate on the LSFBD. In our future work, we will further explore the specific brain inspiration and visual attention mechanism for unconstrained facial beauty prediction task.

## Figures and Tables

**Table 1:** The proposed BeautyNet architecture dimensions.

Name	Filter size/stride, pad	Output size	No. of parameters
Input	—	120 × 120 × 3	—
Conv1	5 × 5/1, 2	120 × 120 × 96	7296
MFM1	—	120 × 120 × 48	—
Pool1	2 × 2/2	60 × 60 × 48	—
Conv2	1 × 1/1	60 × 60 × 96	4704
MFM2	—	60 × 60 × 48	—
Conv3	3 × 3/1, 1	60 × 60 × 192	83136
MFM3	—	60 × 60 × 96	—
Pool3	2 × 2/2	30 × 30 × 96	—
Conv4	1 × 1/1	30 × 30 × 192	18624
MFM4	—	30 × 30 × 96	—
Conv5	3 × 3/1, 1	30 × 30 × 384	332160
MFM5	—	30 × 30 × 192	—
Pool5	2 × 2/2	15 × 15 × 192	—
Conv6	1 × 1/1	15 × 15 × 384	74112
MFM6	—	15 × 15 × 192	—
Conv7	3 × 3/1, 1	15 × 15 × 256	442624
MFM7	—	15 × 15 × 128	—
Conv8	1 × 1/1	15 × 15 × 256	33024
MFM8	—	15 × 15 × 128	—
Conv9	3 × 3/1, 1	15 × 15 × 256	295168
MFM9	—	15 × 15 × 128	—
Pool9	2 × 2/2	8 × 8 × 128	—
Conv10	1 × 1/1, 0	8 × 8 × 512	66048
MFM10	—	8 × 8 × 256	—
Conv11	1 × 1/1	8 × 8 × 2048	526336
MFM11	—	8 × 8 × 1024	—
Res1	—	8 × 8 × 1152	—
Pool12	2 × 2/2	4 × 4 × 256	—
Pool13	2 × 2/2	4 × 4 × 128	—
Pool14	2 × 2/2	4 × 4 × 128	—
Fc1	—	1 × 1 × 512	786944
MFM12	—	1 × 1 × 256	—
Drop1	—	1 × 1 × 256	—
Fc2	—	1 × 1 × 5	1280
Total			2671456

**Table 2:** Classification and Pearson's correlation coefficient results under various numbers of convolution layers.

Proposed network with different depths	Classification accuracy (%)	Pearson's correlation coefficient
NET-5	64.36	79.61
NET-7	64.65	79.20
NET-9	**64.84**	**80.20**
NET-11	62.21	79.05

**Table 3:** Comparison of network parameters using different activation functions.

Proposed network	Activation function	No. of parameters	Storage space (M)	Time (ms/frame)	Classification accuracy (%)
LightenNet	ReLU	1357664	20.9	224.57	62.79
	Sigmoid			234.64	—
	Tanh			233.90	—
	MFM			180.78	64.36

BeautyNet	ReLU	2671461	55.2	287.94	63.48
	Sigmoid			282.18	—
	Tanh			291.10	63.48
	MFM			203.68	64.84

— indicates that the model does not converge.

**Table 4:** Performance comparisons with and without multiscale structures.

Proposed network	Classification accuracy (%)	Pearson's correlation coefficient
LightenNet	64.36	79.41
BeautyNet	64.84	80.20

**Table 5:** Model validation on the LFW database.

Network	DeepFace [41]	DeepID2+ [42]	FaceNet [43]	VGG [44]	Proposed network
Classification accuracy (%)	97.77	97.27	47.1	98.70	99.23

**Table 6:** Prediction accuracy and Pearson's correlation coefficient for transferring different layers.

Transferring proposed network	1	2	3	4	5	Classification accuracy (%)	Pearson's correlation coefficient
Scratch	77.00	29.00	72.00	58.00	56.00	64.84	80.20
Transferring conv1	66.00	39.00	75.00	56.00	55.00	65.92	79.00
Transferring conv2	62.00	34.00	75.00	57.00	50.00	65.82	79.26
Transferring conv3	77.00	30.00	74.00	52.00	53.00	65.52	79.37
Transferring conv4	70.00	27.00	72.00	59.00	56.00	65.53	80.44
Transferring conv5	66.00	40.00	71.00	61.00	58.00	65.92	81.07
Transferring conv6	74.00	25.00	74.00	54.00	58.00	64.90	80.91
Transferring conv7	69.00	36.00	73.00	57.00	55.00	66.02	81.46
Transferring conv8	72.00	37.00	72.00	55.00	51.00	65.23	80.35
Transferring conv9	70.00	25.00	80.00	62.00	39.00	65.82	81.59
Transferring multiscale layer	68.00	32.00	73.00	59.00	62.00	**67.48**	82.96
Transferring Fc1 layer	73.00	28.00	74.00	53.00	54.00	65.53	**83.54**

1, 2, 3, 4, and 5 show the classification accuracy for each specific category.

**Table 7:** Performance comparison of state-of-the-art methods.

Method	Transfer learning	Classification accuracy (%)	Pearson's correlation coefficient
Raw pixel [15]	No	48.11	—
Eigenfaces [15]	No	46.52	—
CRBM [15]	No	51.62	—
K-means [15]	No	52.54	—
Multiscale K-means [15]	No	55.07	—
NIN_Imagenet [45]	No	55.60	74.69
NIN_Imagenet [45]	Yes	58.30	76.96
DeepID2 [46]	No	55.90	73.75
DeepID2 [46]	Yes	60.25	76.57
GoogLeNet [47]	No	57.20	78.14
GoogLeNet [47]	Yes	62.59	79.78
VGG_CNN_S [48]	No	57.30	78.87
VGG_CNN_S [48]	Yes	62.69	81.12
LightenNet	No	64.36	79.41
LightenNet	Yes	65.82	81.59
BeautyNet	No	64.84	80.20
BeautyNet	Yes	**67.48**	**83.54**

## Data Availability

The data used to support the findings of this study are available from the corresponding author upon request.

## References

[1] Laurentini A., Bottino A. (2014). Computer analysis of face beauty: a survey. *Computer Vision and Image Understanding*.

[2] Taleb A., Songyao J., Yun F. Rule-based facial makeup recommendation system.

[3] Liu Y., Xie Z., Yuan X., Chen J., Song W. (2017). Multi-level structured hybrid forest for joint head detection and pose estimation. *Neurocomputing*.

[4] Lamyaa S., Taoufiq G., Hassan E. (2018). A novel deep learning approach for recognizing stereotypical motor movements within and across subjects on the autism spectrum disorder. *Computational Intelligence and Neuroscience*.

[5] Yin W., Kann K., Yu M. (2017). Comparative study of CNN and RNN for natural language processing.

[6] Zhang K., Liu Q., Wu Y. (2016). Robust visual tracking via convolutional networks without training. *IEEE Transactions on Image Processing*.

[7] Blanco P. J., Sánchez P. J., de Souza Neto E. A., Feijóo R. A. (2014). Variational foundations and generalized unified theory of RVE-based multiscale models. *Archives of Computational Methods in Engineering*.

[8] Wancun L., Wenyan T., Liguo Z. Multi-scale behavior learning for multi-object tracking.

[9] Pan S. J., Yang Q. (2010). A survey on transfer learning. *IEEE Transactions on Knowledge and Data Engineering*.

[10] Weiss K., Khoshgoftaar T. M., Wang D. (2016). A survey of transfer learning. *Journal of Big Data*.

[11] Mao H., Jin L., Du M. Automatic classification of Chinese female facial beauty using Support Vector Machine.

[12] Zhang D., Zhao Q., Chen F. (2011). Quantitative analysis of human facial beauty using geometric features. *Pattern Recognition*.

[13] Gunes H., Piccardi M., Jan T. Comparative beauty classification for pre-surgery planning.

[14] Schölkopf B., Platt J., Hofmann T. A humanlike predictor of facial attractiveness.

[15] Zhai Y., Huang Y., Xu Y. Benchmark of a large scale database for facial beauty prediction.

[16] Gray D., Yu K., Xu W. Predicting facial beauty without landmarks.

[17] Gan J., Li L., Zhai Y., Liu Y. (2014). Deep self-taught learning for facial beauty prediction. *Neurocomputing*.

[18] Xu J., Jin L., Liang L. (2015). A new humanlike facial attractiveness predictor with cascaded fine-tuning deep learning model. *Computer Science*.

[19] Chen F., Zhang D., Wang C. Comparison and fusion of multiple types of features for image-based facial beauty prediction.

[20] Xu J., Jin L., Liang L. Facial attractiveness prediction using psychologically inspired convolutional neural network (PI-CNN).

[21] Zhang D., Chen F., Xu Y. (2018). Data-driven facial beauty analysis: prediction, retrieval and manipulation. *IEEE Transactions on Affective Computing*.

[22] Wang Y., Luo Z., Xu Z. Fusion of infrared and visual images through multiscale hybrid unidirectional total variation.

[23] Xie S., Tu Z. Holistically-nested edge detection.

[24] Liu J., Zha Z. J., Tian Q. I. Multi-scale triplet CNN for person re-identification.

[25] Yang S., Ramanan D. Multi-scale recognition with DAG-CNNs.

[26] Li G., Yu Y. Visual saliency based on multiscale deep features.

[27] Ma C., Huang J. B., Yang X., Yang M. H. Hierarchical convolutional features for visual tracking.

[28] Zhao Z., Xu G., Qi Y. Multi-Scale Hierarchy deep feature aggregation for compact image representations.

[29] Faraji M. R., Qi X., Qi X. J. (2018). Face recognition under varying illuminations with multi-scale gradient maximum response. *Neurocomputing*.

[30] Lu Z., Zhu Y., Pan S. J. Source free transfer learning for text classification.

[31] Zhao L., Xiang E. W., Zhong E. Active transfer learning for cross-system recommendation.

[32] Zhu Y., Chen Y., Lu Z. Heterogeneous transfer learning for image classification.

[33] Yosinski J., Clune J., Bengio Y., Lipson H. (2014). How transferable are features in deep neural networks. *Eprint Arxiv*.

[34] Shelhamer E., Long J., Darrell T. (2017). Fully convolutional networks for semantic segmentation. *IEEE Transactions on Pattern Analysis and Machine Intelligence*.

[35] Shin H.-C., Roth H. R., Gao M. (2016). Deep convolutional neural networks for computer-aided detection: CNN architectures, dataset characteristics and transfer learning. *IEEE Transactions on Medical Imaging*.

[36] Girshick R., Donahue J., Darrell T., Malik J. (2016). Region-based convolutional networks for accurate object detection and segmentation. *IEEE Transactions on Pattern Analysis and Machine Intelligence*.

[37] Wang J., Yuan C. Facial expression recognition with multi-scale convolution neural network.

[38] Cai Z., Fan Q., Feris R. S. A unified multi-scale deep convolutional neural network for fast object detection.

[39] Wu Y., Jia N., Sun J. (2014). Real-time multi-scale tracking based on compressive sensing. *The Visual Computer*.

[40] Jia Y., Shelhamer E., Donahue J. Caffe: convolutional architecture for fast feature embedding.

[41] Taigman Y., Yang M., Ranzato M. DeepFace: closing the gap to human-level performance in face verification.

[42] Sun Y., Wang X., Tang X. Deeply learned face representations are sparse, selective, and robust.

[43] Schroff F., Kalenichenko D., Philbin J. Facenet: a unified embedding for face recognition and clustering.

[44] Fan H., Cao Z., Jiang Y. (2014). Learning deep face representation.

[45] Lin M., Chen Q., Yan S. (2013). Network in network.

[46] Sun Y., Chen Y., Wang X. Deep learning face representation by joint identification-verification.

[47] Szegedy C., Liu W., Jia Y. Going deeper with convolutions.

[48] Simonyan K., Zisserman A. (2014). Very deep convolutional networks for large-scale image recognition.

